# Patient-reported outcomes with hypoglossal nerve stimulation for treatment of obstructive sleep apnea: a systematic review and meta-analysis

**DOI:** 10.1007/s00405-023-08062-1

**Published:** 2023-06-24

**Authors:** Marcel Braun, Manuel Stoerzel, Mathias Wollny, Christoph Schoebel, J. Ulrich Sommer, Clemens Heiser

**Affiliations:** 1 Department of Pneumology, University Medicine Essen – Ruhrlandklinik, West German Lung Center, University Duisburg-Essen, Duisburg, Germany; 2 Faculty of Sleep and Telemedicine, University Medicine Essen – Ruhrlandklinik, West German Lung Center, University Duisburg-Essen, Tueschener Weg 40, 45239 Essen, Germany; 3 German Cancer Research Center (DKFZ), Heidelberg, BW Germany; 4 MedImbursement, Tarmstedt, Germany; 5 Department of Otorhinolaryngology/Head and Neck Surgery, Klinikum rechts der Isar, Technical University of Munich, Munich, Germany; 6 ENT-Center Mangfall-Inn, Bad Aibling, Germany

**Keywords:** Quality of life, Sleep-disordered breathing, Sleep surgery, Technology assessment

## Abstract

**Introduction:**

Hypoglossal nerve stimulation (HNS) has recently been introduced as an alternative treatment for patients with OSA. A large number of studies have demonstrated substantial changes in OSA with this therapy by reducing respiratory events and improving symptoms such as daytime sleepiness and quality of life. The objective of this review was to conduct a systematic review and meta-analysis to evaluate patient-reported outcomes and experience with HNS therapy.

**Methods:**

A systematic literature search of MEDLINE, Cochrane, and Web of Science was performed to identify randomized controlled and observational studies reporting subjective outcomes with different HNS systems in patients with OSA. Abstracts of 406 articles were screened and a subset of 55 articles were reviewed for eligibility. Risk of bias was assessed using the ROBINS-I tool. Meta-analysis using RevMan was performed when > 2 studies were identified that reported data on a specific outcome.

**Results:**

Thirty-four publications reporting data on 3785 patients with a mean follow-up of 11.8 ± 12.2 months were identified and included in the meta-analysis. The analysis revealed a pooled effect of 4.59 points improvement in daytime sleepiness as measured by the ESS questionnaire (Z = 42.82, *p* < .001), 2.84 points improvement in daytime functioning as measured by the FOSQ score (Z = 28.38, *p* < .001), and 1.77 points improvement in sleep quality as measured by the PSQI questionnaire (Z = 2.53, *p* = .010). Patient-reported experience was consistently positive and revealed additional relevant aspects from this perspective.

**Conclusion:**

HNS therapy significantly improves quality of life in patients with OSA and reliably produces clinically meaningful effects on daytime sleepiness, daytime functioning, and sleep quality. Treatment regularly meets or exceeds the minimum clinically important differences defined for the respective instruments. Additional research is needed to further investigate effects on quality of life beyond improvements in daytime sleepiness and daytime functioning.

**Supplementary Information:**

The online version contains supplementary material available at 10.1007/s00405-023-08062-1.

## Introduction

Among respiratory disorders, obstructive sleep apnea (OSA) is one of the most common, potentially affecting up to a quarter of the world’s population [1–3]. Although highly prevalent, the condition is often unrecognized until patients report symptoms such as sleep disruption and insomnia, daytime sleepiness, reduced daytime functioning, or associated neurocognitive disorders. In addition, if left untreated, OSA can lead to a variety of cardiovascular, neurological, and metabolic comorbidities [4, 5]. Due to difficulties in maintaining wakefulness during the day, patients with untreated OSA are more likely to be involved in motor vehicle collisions or occupational accidents [6, 7]. Recently, OSA has been increasingly recognized as a prognostic factor in cancer [8, 9].

OSA is considered to be a multifactorial disease that leads to collapse of the soft tissues of the upper airway during sleep when muscle tone decreases [4]. Besides advanced age, smoking and alcohol consumption, obesity is the main risk factor for developing OSA, although anatomical factors such as retrognathia also increase the likelihood of developing OSA.

From a patient perspective, several dimensions of OSA treatment have been identified as outcome-relevant, although their use in clinical research and routine practice is highly heterogeneous [10]. A core outcome set for effectiveness research in OSA, which will harmonize outcome-relevant endpoints, is currently under development [11]. From various studies, patient preferences in OSA treatment are relatively well understood and are increasingly considered relevant to improve the effectiveness of health interventions, which often depends on adherence. As such, high clinical efficacy, i.e., reduction in the risk of OSA-related comorbidities, improvements in daytime sleepiness and fatigue, and low rates of treatment side effects and adverse events are preferred from the patient's perspective [12–14].

To date, there is no curative treatment for OSA. In clinical practice, various therapies are used to prevent collapse of the upper airway muscles during sleep [15, 16]. In most countries, positive airway pressure (PAP) therapy is used as a first-line treatment, which is highly effective in reducing obstructive events and improving symptoms when used consistently. [However, many patients with OSA have difficulty adhering to PAP therapy due to side effects, complications, and impaired sleep quality [17–19]. Various treatments have been introduced as alternatives for patients who cannot tolerate PAP and are routinely used in clinical practice. In the non-surgical field, mandibular advancement devices (MAD) and position trainers are recommended in treatment guidelines. Among surgical therapies, resecting procedures such as tonsillectomy or soft palate surgery are used in selected patients [20]. Recently, hypoglossal nerve stimulation (HNS), which uses electrical stimulation to activate the upper airway dilator muscles at night, has been introduced as a new treatment option [21]. Several studies and real-world analyses have shown that HNS can lead to a sustained reduction in obstructive events and improve sleep-related quality of life in patients with appropriate airway anatomy and low body mass index [22–25]. In addition to observational cohort studies, randomized controlled trials and meta-analyses have underscored the efficacy of HNS therapy [26–30]. A recently published study comparing PAP therapy with HNS using propensity score stratification showed non-inferiority in reducing obstructive events and superiority in improving sleep-related quality of life, one of the most important patient-relevant endpoints in OSA [31].

Patient-reported outcome and patient-reported experience (PROM, PREM) measures have become increasingly important in the health sciences over the past decade, particularly in the development and evaluation of new medical technologies during market introduction. In addition to generic instruments for reporting quality of life and subjective health status, such as the Short Form 36 (SF-36) or the European Quality of Life Questionnaires (EQ-5D), indication-specific instruments are an important element for determining disease severity and for assessing the efficacy and effectiveness of health interventions. PROM, and increasingly PREM, are commonly reported as endpoints in clinical trials, but are also often part of regulatory submissions for new medical products [32, 33]. In sleep medicine, several PROM have been validated and are commonly used in clinical practice to assess sleep-related quality of life [34]. Since a core outcome set for OSA hasn’t been implemented yet, there is a high variability of instruments used in clinical trials and routine practice.

Although numerous studies have been published on the efficacy and effectiveness of HNS, no attempt has been made to quantify the effect size of PROM across these publications. Therefore, the aim of this study is to conduct a systematic review and aggregate the effects of PROM and PREM in a meta-analysis.

## Methods

The research question was defined a priori using the PICO format. For the purpose of this analysis, any study that evaluated adult patients with OSA treated with HNS therapy and reported changes between baseline and follow-up on any PROM or PREM was included.

### Data collection, quality assessment and extraction

Based on the research question, a search strategy was developed to identify publications of interest and searches were conducted in MEDLINE, EMBASE, Cochrane, and Google Scholar. The search included studies published up to August 30, 2022. Data were collected electronically using Rayyan software [35]. As a first step, two researchers (MB, MW) independently screened articles for eligibility, blinded to each other and to the researcher who performed the searches (MS) to reduce identification bias. Screening included an initial review of abstracts for inclusion and exclusion criteria. These were defined as follows:

- Inclusion criteria: Publication 01/2000–08/2022, publication in English language, ≥ 10 subjects included, follow-up ≥ 3 months; reporting PROM or PREM in OSA-relevant outcome domain.
- Exclusion criteria: Review articles, case reports, animal or in vitro studies, editorials, abstracts, publications reporting on pediatric populations, publications in languages other than English.

In cases where abstracts did not provide sufficient information, full texts were reviewed to determine eligibility. Discrepancies in screening were discussed among the reviewers until consensus was reached. The screening process was documented according to the Preferred Reporting Items for Systematic Review and Meta-analysis (PRISMA) framework [36].

Per PICO question for this analysis, inclusion of research from both randomized controlled trials and observational cohort studies was allowed to achieve a comprehensive understanding of PROM in HNS. To assess the quality of the included studies, a risk of bias assessment was performed according to the Cochrane Handbook for Systematic Reviews of Interventions using the ROBINS-I tool for observational and case–control studies [37, 38].

After a final set of articles was identified for inclusion in the meta-analysis, PROM data were extracted into an MS Excel database. Extracted information included PROM instrument, study type and stimulation method studied, mean and standard deviation of PROM data, and follow-up period.

### Statistical analysis

Patient-reported outcome data were extracted as mean ± standard deviation and entered into RevMan 5.4 for meta-analysis (Review Manager, version 5.4, Copenhagen/Denmark). Effect sizes were reported as the mean difference in change from baseline to the last reported follow-up with a 95% confidence interval (CI). Due to the strict labeling of HNS therapy, resulting in a fairly homogeneous patient population, and the fact that the purpose of the study is to aggregate outcomes in this particular population, a fixed effects model was considered appropriate and selected for analysis. A Chi^2^ test and Higgins and Thompson's I^2^ statistics were used to assess heterogeneity among the included studies. Results were considered statistically significant at an alpha level of 0.05 for two-tailed z-tests.

Welch's tests were used to assess differences between stimulation methods, which were considered of interest due to the different underlying mechanisms of action of the HNS stimulation technologies included in this study.

### Publication bias

Publication bias was tested using Egger’s regression analysis when three or more studies were identified for an outcome [39, 40]. *P*-values of < 0.05 were considered significant for presence of bias, and trim and fill adjusted analysis conducted to remove outliers from the positive side of the funnel plot, and evaluate the revised effect size.

## Results

### Research identification and quality assessment

**Fig. 1:**
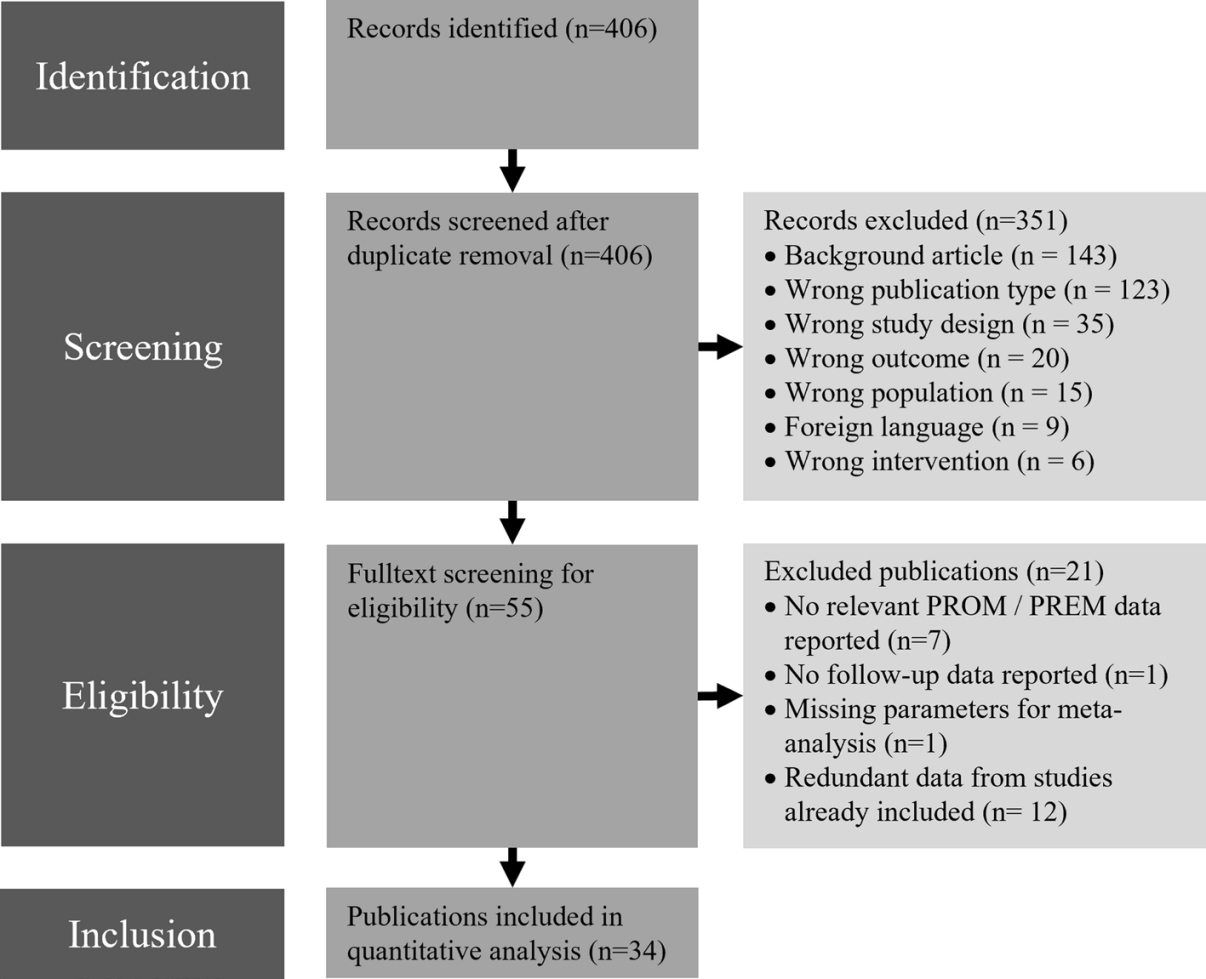
PRISMA flow diagram of systematic review

From the a priori defined literature search, 406 studies were identified and their abstracts were screened by two independent investigators using Rayyan software [35]. Of these 406 articles, 351 had to be excluded for various reasons, as shown in the PRISMA diagram, and 55 articles were included for full-text screening to determine eligibility for inclusion in the meta-analysis (Fig. 1). Twenty-one had to be excluded because they used data from studies already included (n = 12), did not report relevant PROM or PREM data (n = 7), did not include follow-up data (n = 1), or lacked data required for meta-analysis (n = 1). The final set included for meta-analysis consisted of 34 studies reporting data on a total of 3785 patients with a mean follow-up of 11.8 ± 12.2 months. Thirty-one studies with 3701 patients reported on respiration-synchronized HNS (mean follow-up = 12.2 ± 12.8 months), while three studies with a total of 84 patients reported on continuous HNS therapy (mean follow-up = 8.0 ± 2.8 months) (Tables 1 and 2).

**Table 1:** PROM instruments used in included research articles

Instrument	Outcome domain	Scale	Direction	Minimal important difference	References
Epworth Sleepiness Scale (ESS)	Assessment of daytime sleepiness in OSA	0–24	↑	2.0 points	[41, 42]
Functional Outcomes of Sleep Questionnaires (FOSQ)	Impairment of daytime activities due to sleepiness or fatigue	5–20	↓	1.8 points	[43, 44]
Fatigue Severity Scale (FSS)	Impact of fatigue	1–7	↑	0.45 points	[45, 46]
Pittsburgh Sleep Quality Index (PSQI)	Sleep quality and sleep disturbances	0–21	↑	4.4 points	[47, 48]
Calgary Sleep Apnea Quality of Life Index (SAQLI)	Impairment of different functions due to sleep apnea	0–5	↓	1.0 points	[49, 50]
Insomnia Severity Index (ISI)	Assessment of severity and impact of insomnia	0–28	↑	6.0 points	[51, 52]
Patient Health Questionnaire (PHQ-9)	Quantify depression symptoms and monitor severity	0–27	↑	5.0 points	[53, 54]

↑ = Higher scores indicating larger negative effects, ↓ = Lower scores indicating smaller negative effects

A researcher not involved in the screening and eligibility process (MS) performed the quality assessment using the ROBINS-I tool for observational and case–control studies. The majority of included studies were found to have low/moderate bias, and only five and two were found to have severe and critical bias, respectively (Table 2 and full assessment in Table 1 in the online supplement).

**Table 2:** Overview of studies included for meta-analysis

Study	Year	Sample size	Follow-up duration (months)	Included PROM instruments	Study design	Stimulation method	Overall bias	References
Baptista et al.	2021	18	3	ESS	Observational cohort study	Breathing-synchronized	● Critical	[41]
Eastwood et al.	2011	21	6	ESS, FOSQ, PSQI, SAQLI	Observational cohort study	Breathing-synchronized	● Serious	[58]
Eastwood et al.	2019	27	6	ESS, FOSQ	Observational cohort study	Continuous	● Moderate	[59]
Friedman et al.	2016	43	6	ESS, SAQLI	Observational cohort study	Continuous	● Moderate	[60]
Heiser et al.	2017	31	12	ESS	Observational cohort study	Breathing-synchronized	● Moderate	[61]
Heiser et al.	2021	89	30	ESS	Randomized-controlled trial	Breathing-synchronized	● Low	[27]
Heiser et al.	2022	227	12	ESS	Propensity-score comparison	Breathing-synchronized	● Low	[31]
Hinder et al.	2022	50	12	ESS	Observational cohort study	Breathing-synchronized	● Moderate	[62]
Hofauer et al.	2017	26	3	ESS	Observational cohort study	Breathing-synchronized	● Moderate	[63]
Hofauer et al.	2019	102	36	ESS	Observational cohort study	Breathing-synchronized	● Low	[39]
Huntley et al.	2018	164	3	ESS	Observational cohort study	Breathing-synchronized	● Low	[64]
Kent et al.	2019	584	12	FOSQ	Observational cohort study	Breathing-synchronized	● Low	[65]
Kent et al.	2016	20	3	ESS	Observational cohort study	Breathing-synchronized	● Serious	[66]
Kezirian et al.	2014	31	12	ESS, PSQI	Observational cohort study	Breathing-synchronized	● Moderate	[67]
Kumar et al.	2019	114	3	ESS	Observational cohort study	Breathing-synchronized	● Low	[68]
Mahmoud et al.	2018	47	3	ESS	Observational cohort study	Breathing-synchronized	● Moderate	[69]
Mwenge et al.	2013	14	12	ESS, FSS	Observational cohort study	Continuous	● Moderate	[71]
Parikh et al.	2018	14	12	ESS	Observational cohort study	Breathing-synchronized	● Serious	[72]
Pascoe et al.	2022	85	12	ESS, FOSQ, ISI, PHQ-9	Observational cohort study	Breathing-synchronized	● Moderate	[42]
Patil et al.	2021	46	3	ESS	Observational cohort study	Breathing-synchronized	● Moderate	[73]
Patil et al.	2021	53	12	ESS	Observational cohort study	Breathing-synchronized	● Moderate	[74]
Pawlak et al.	2021	56	6	ESS	Observational cohort study	Breathing-synchronized	● Moderate	[75]
Philip et al.	2018	10	6	ESS	Observational cohort study	Breathing-synchronized	● Critical	[76]
Sarber et al.	2020	31	3	ESS	Observational cohort study	Breathing-synchronized	● Moderate	[77]
Sarber et al.	2020	18	6	ESS	Observational cohort study	Breathing-synchronized	● Serious	[78]
Shah et al.	2018	40	3	ESS	Observational cohort study	Breathing-synchronized	● Moderate	[80]
Steffen et al.	2019	25	24	ESS	Observational cohort study	Breathing-synchronized	● Moderate	[81]
Steffen et al.	2020	60	36	ESS, FOSQ	Observational cohort study	Breathing-synchronized	● Moderate	[25]
Suurna et al.	2021	1019	12	ESS	Observational cohort study	Breathing-synchronized	● Moderate	[70]
Van de Heyning et al.	2012	28	6	ESS	Observational cohort study	Breathing-synchronized	● Moderate	[21]
Weeks B et al	2018	18	3	ESS	Observational cohort study	Breathing-synchronized	● Serious	[82]
Withrow et al.	2019	600	12	ESS	Observational cohort study	Breathing-synchronized	● Low	[87]
Woodson et al.	2018	126	36	ESS, FOSQ	Observational cohort study	Breathing-synchronized	● Low	[24]
Zhu et al.	2018	62	12	ESS	Observational cohort study	Breathing-synchronized	● Moderate	[88]

### Identified patient-relevant outcomes measures

Seven different PROM instruments were identified from the included studies. All 34 studies used the ESS as a measure of daytime sleepiness in OSA patients, seven reported changes in daytime functioning as measured by the FOSQ. The SAQLI was used in three studies, the PSQI in two studies, and the ISI, FSS, and PHQ-9 in one study each.

### Epworth sleepiness scale

**Fig. 2:**
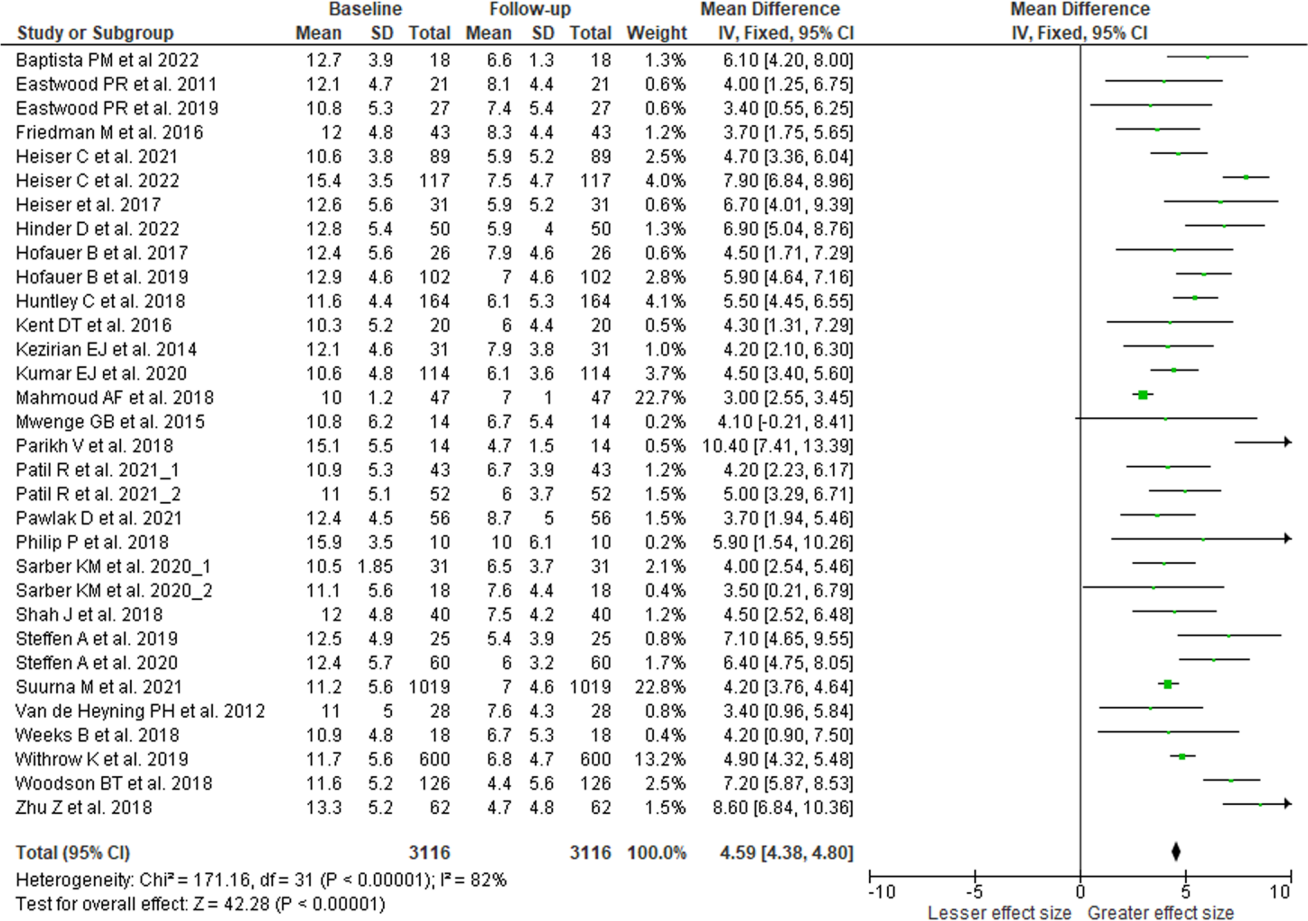
Forest plot of changes in daytime sleepiness with HNS therapy, measured with Epworth Sleepiness Scale (ESS), reduction of ESS scores indicates greater symptom improvement

Quantitative analysis to assess change in daytime sleepiness as measured by the ESS questionnaire used a fixed effects model (*p* < 0.001, I^2^ = 82%) and showed a pooled effect of 4.59 points improvement on the ESS questionnaire (95% CI 4.38–4.80; Z = 42.82, *p* < 0.001; Fig. 2). One study was removed because it included ESS data from cohorts reported in other articles. All studies included in the analysis, which included data from 3,116 subjects, reported changes in ESS scores that met or exceeded the MID of 2.0 points improvement. Breath-synchronized stimulation had a mean improvement of 4.61 points (95% CI 4.39–4.82; Z = 42.03, *p* < 0.001), while continuous stimulation decreased ESS scores by a mean of 3.61 points (95% CI 2.16–5.17; Z = 4.77, *p* < 0.001). A two-sample Welch t-test showed that the mean ESS improvement was significantly greater with breathing-synchronized stimulation compared to continuous stimulation (t (18.0) = 3.92, *p* < 0.001).

### Functional outcomes of sleep questionnaire

**Fig. 3:**
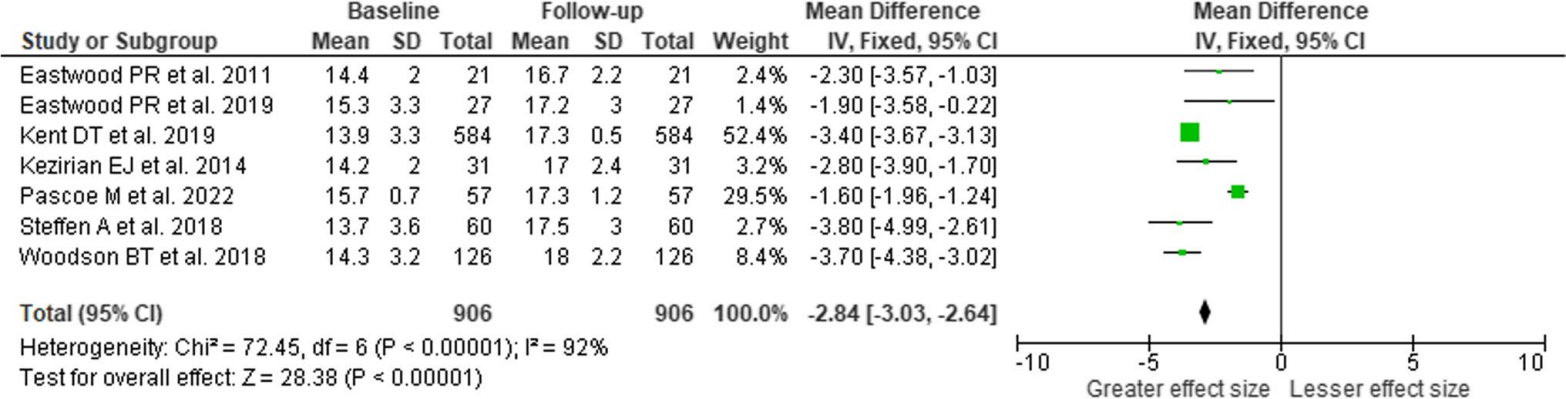
Forest plot on effects of HNS therapy on daytime functioning, measured with Functional Outcomes of Sleep Questionnaire (FOSQ), increase of FOSQ scores indicates greater symptom improvement

Data on the FOSQ instrument to assess changes in daytime functioning were available from seven studies, reporting results for 906 patients (Fig. 3). Following the rationale above, a fixed effects model was used to aggregate the results. There was substantial heterogeneity with an I^2^ of 92% (*p* < 0.001). A pooled effect size of 2.84 points improvement in FOSQ score (95% CI 2.64–3.03; Z = 28.38, *p* < 0.001). Due to the relatively small number of studies reporting FOSQ data, a comparative analysis by stimulation method could not be performed.

### Calgary sleep apnea quality of life index (SAQLI)

**Fig. 4:**
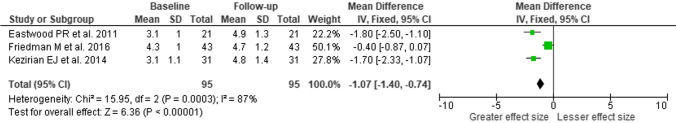
Forest plot on effects of HNS therapy on daytime functioning, measured with Calgary Sleep Apnea Quality of Life Index (SAQLI), increase of SAQLI scores indicates greater symptom improvement

The SAQLI questionnaire, which assesses the impact of OSA on five dimensions of daily life, was used in three studies with a total of 95 subjects (Fig. 4). In the meta-analysis using a fixed effects model, a mean difference of 1.07 (95% CI 0.74–1.40; Z = 6.36, p < 0.001) was calculated, which met the minimal important difference of 1.0 points. Again, a comparative analysis by stimulation method was not performed due to the small number of studies identified.

### Pittsburgh sleep quality index (PSQI)

**Fig. 5:**
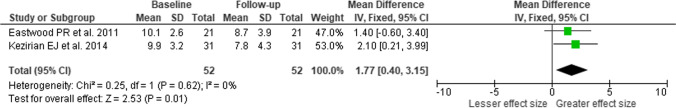
Forest plot on effects of HNS therapy on sleep quality, measured with Pittsburgh Sleep Quality Index (PSQI), reduction of PSQI scores indicates greater symptom improvement

For the PSQI, two studies were identified that reported a mean change of 1.77 (95% CI 0.44–3.15; Z = 2.53, *p* = 0.010). The minimal important difference for this instrument of 4.0 points was not reached (Fig. 5).

### Other patient-reported outcome measurements

A few other PROM instruments were identified in the systematic literature review, but could not be included in the meta-analysis because only one study per PROM was identified. To provide a complete overview of patient-reported outcomes with HNS therapy, these are reported individually in Table 3. Of the three PROMs identified, two met the minimum important difference.

**Table 3:** Effects of HNS therapy on self-reported insomnia, depression, and fatigue

Instrument	Clinical dimension	N	Baseline (mean ± SD)	Follow-up (mean ± SD)	Follow-up duration (months)	MID/MID reached	References
Insomnia Severity Index (ISI)	Insomnia	62	15.16 ± 1.46	10.46 ± 2.24	12	6.0/no	[70]
Patient Health Questionnaire (PHQ-9)	Depression	29	8.11 ± 1.31	4.90 ± 1.90	12	3.7/no	[70]
Fatigue Severity Scale (FSS)	Fatigue	13	4.50 ± 1.60	3.60 ± 1.50	12	0.45/yes	[68]

*SD* standard deviation, *MID* minimal important difference

### Patient-reported experience measures

As there is no standardized instrument to assess patient experience with HNS therapy, a qualitative review was conducted. Of the 34 articles included from the literature search, three reported on patient experience, including attitudes toward HNS therapy, satisfaction with treatment, and comparison with PAP ventilation. Hofauer et al. described overall positive attitudes towards HNS therapy in the areas of improvement of sleep-related and general health and quality of life, realization of expectations and satisfaction with the treatment decision [39]. Suurna et al. reported on the experience of 1016 patients enrolled in the ADHERE registry using a questionnaire introduced by Hasselbach et al. in 2018 [55, 70]. Here, 91% of patients were satisfied with HNS therapy, 92% would choose the therapy again and 94% would recommend it. 92% considered the treatment to be superior to PAP ventilation used prior to stimulation therapy. The same questionnaire was used by Baptista et al. in a cohort of patients treated in Spain, for which comparable experiences were reported with values of 86%, 89%, 89% and 91%, respectively [41].

### Publication bias assessment

Egger’s test for publication bias was significant for the outcome ESS (p = 0.020). Sensitivity analyses, in which studies expected on the negative side of the funnel plot were imputed by using the trim and fill method were included, showed no significant differences in effect size (4.59, points, 95% CI 4.38–4.80 points; vs. 4.06 points, 95% CI 3.86–4.26). There was no evidence of publication bias for the outcomes FOSQ (p = 0.984) and SAQLI (p = 0.136), and the outcome PSQI was not evaluated, since only two studies were included in the analysis.

## Discussion

Alternative treatment options for patients with OSA who cannot tolerate PAP therapy have been limited for many years. Recently, the range of options has expanded significantly, improving the management of this serious chronic condition that affects a large number of patients worldwide. Nocturnal hypoglossal nerve stimulation has been shown in a number of studies to reduce the number of respiratory events during sleep and improve sleep-related quality of life. While most of the evidence is based on observational studies, randomized controlled trials and meta-analyses have also been conducted. This study is the first to report patient-reported outcomes and experiences in a systematic review and meta-analysis. Subjective outcomes of HNS therapy were considered particularly important because objective measures of sleep apnea, such as the Apnea Hypopnea Index (AHI), do not always correlate with OSA symptoms such as daytime sleepiness and impaired sleep quality [42–44]. Furthermore, as a chronic condition with a significant impact on quality of life, patient-reported outcomes and treatment experience are important factors influencing treatment acceptance and adherence, which is of great importance given the nature of an implant-based treatment that requires surgical intervention.

The current first-line treatment for OSA, PAP ventilation, has been shown in numerous studies to be effective in reducing respiratory events and improving symptoms such as daytime sleepiness and daytime functioning [45–47]. Long-term adherence is often low due to side effects and complications [17, 48].

The ESS questionnaire, which is the dominant tool in sleep apnea outcome research, was reported in all studies included in this analysis. Regardless of the stimulation method used, HNS therapy consistently reduces OSA daytime sleepiness as measured by the ESS beyond the minimally important difference and sustainably for up to three to five years of follow-up. Though we summarized outcomes of different stimulation systems here, it is important to highlight that differences between them were found for certain domains of interest, such as improvement of daytime sleepiness, measured with the ESS questionnaire.

The magnitude of ESS improvement summarized in this systematic review and meta-analysis is greater than the changes commonly observed with PAP therapy [86]. Though it was not the objective of this study to compare the two methods, it is an interesting finding that is consistent with current research comparing HNS therapy to PAP ventilation, which shows superior efficacy in improving symptoms of OSA with nocturnal stimulation [31, 50].

Another important dimension of patient-reported outcomes is daytime functioning, which is often impaired by poor and non-restorative sleep in OSA. The FOSQ is a widely used instrument to measure the level of impairment in daytime functioning and was reported in seven studies included in this analysis. Changes in the FOSQ were observed in all studies, and the average improvement of 2.84 points was above the minimally important difference for this questionnaire. The overall magnitude of effect was smaller for the assessment of daytime sleepiness than for the ESS, which may be explained by the broader outcome domains assessed by the FOSQ.

Beyond these two questionnaires, few studies have used other instruments, which represents an opportunity for future research in the area of patient-reported outcomes with HNS therapy. An important area in this regard is OSA-related insomnia, which is often present in patients due to arousals after respiratory events. Changes in this parameter with HNS therapy were identified in only two studies in the literature search for this analysis. A meta-analysis could not be performed due to missing data in one article. Pascoe et al. report significant improvements on the Insomnia Severity Scale (ISI), with 46.9% of 85 patients reaching the MID [50]. In another study published after the literature search was completed, Pordzik et al. confirmed these findings in a cohort of 27 patients who experienced a mean improvement of 5.0 points on the ISI questionnaire [51].

The effect of HNS therapy on depression, one of the most common symptoms in patients with OSA, has also been understudied. For the systematic review of this study, only one article was identified that reported changes in the PHQ-9 questionnaire [42]. Among 48 patients for whom data were available, an average improvement of 4.0 points was reported, with 29.2% reaching the MID for this instrument.

PREMs are an emerging area in outcomes research because they allow evaluation of consequences for patients beyond changes in symptoms. In addition, PREM allow the assessment of the process quality of health care interventions, which is important for estimating the global effects of treatments. Experiences with HNS therapy have been reported to be largely positive across studies, with high levels of satisfaction with treatment and subjective efficacy. The development of a standardized PREM tool for HNS therapy would be beneficial to allow standardized evaluation and comparison across different cohorts and stimulation systems.

## Limitations

Firstly, this study was not registered ex ante at the PROSPERO database of the National Institute for Health and Care research, which would have increased the transparency of the research conducted.

It is also important to emphasize that this meta-analysis included mainly observational cohort or case–control studies and only two studies with randomized controlled trial (RCT) data. Additional RCT data would be valuable for a thorough assessment of outcomes. Also, the aim of this study was to evaluate patient-reported outcomes with HNS therapy, which inherently introduces a bias in the reporting of outcomes. Nevertheless, consideration of the patient perspective is an essential part of health technology assessment worldwide.

Another limitation is the range of outcome domains included in this analysis. These represent only a subset of the potential benefits of OSA treatment in general and HNS treatment in particular. While the ESS is widely used to measure symptoms in patients with OSA, it is important to highlight that recent research identified significant limitations of this tool in the form of greater test–retest variability as initially reported and a larger variance in certain subpopulations [83].

Another limitation is that the study does not include characteristics of the study populations that were included in the meta-analysis, such as gender or age distribution, and which could have an impact on outcomes. In addition, the study did not include adverse events and treatment complications, which are highly relevant from a patient perspective [52]; however, aggregated event rates have been previously reported in detail in meta-analyses by Kompelli et al. and Costantino et al. [28, 29] A recent study by Bellamkonda et al. analyzed adverse events reported to the MAUDE database at the United States Food and Drug Administration, which is a valuable source for real-world data. and found various complications and side-effects, mainly related to the surgical procedure [84]. Those potential events and their likelihood should be considered by patients and physicians in the decision-making process.

Of note, a recent study by Crossley et al. reported a potential bias in HNS studies, since research has been sponsored by manufacturers of the stimulation devices [85]. Though this imposes a conflict of interest, it is common for early clinical research in absence of sufficient funding for these activities.

Finally, most articles reported data on breath-synchronized HNS therapy, including the two RCTs, whereas only four continuous stimulation studies could be identified, which may have biased the results. However, as almost all included studies achieved the MID for the respective devices, the risk is considered to be low.

## Conclusion

The effects of HNS therapy result in significant and sustained improvements in patient-reported outcomes. Changes in the ESS questionnaire, which assesses daytime sleepiness due to OSA, reach the MID and are greater than improvements commonly reported with PAP therapy. The meta-analysis also found significant improvements in other outcome domains, such as daytime functioning and subjective sleep quality, although these did not always reach the MID. Patient-reported experiences with HNS therapy are positive and show high satisfaction with this treatment in several aspects. Thus, HNS therapy is well accepted by patients and leads to significant and clinically meaningful improvements in self-reported QoL.

## Supplementary Information

Below is the link to the electronic supplementary material.

Supplementary file1 (DOCX 385 KB)

## Data Availability

The data of this study are available from the corresponding author upon reasonable request.
