# Supplementary material for: Patient-reported outcomes with hypoglossal nerve stimulation for treatment of obstructive sleep apnea: a systematic review and meta-analysis
Source: Eur Arch Otorhinolaryngol. 2023 Jun 24;280(10):4627–39. doi: 10.1007/s00405-023-08062-1 (PMC10477259; doi:10.1007/s00405-023-08062-1)
Supplement: Supplementary file 1 — Supplementary file1 (DOCX 385 KB) [file 405_2023_8062_MOESM1_ESM.docx]

**Supplement to**

**Patient-reported Outcomes with Hypoglossal Nerve Stimulation for Treatment of Obstructive Sleep Apnea:**

**A systematic review and Meta-analysis**

1. **Search strategy**
   1. **Key words**

“Obstructive sleep apnea[Title/Abstract] OR Upper airway resistance[Title/Abstract]) AND (Implantable nerve stimulator[Title/Abstract] OR hypoglossal[Title/Abstract] OR Inspire implant[Title/Abstract]”

- 1. **Inclusion & exclusion criteria**

| **Inclusion criteria** | **Exclusion** |
| --- | --- |
| Publication between 01/2000 and 08/2022 | Review articles |
| English language publication | Animal studies or in-vitro studies |
| At least ten subjects included in study | Editorials |
| Follow-up period at least 3 months | Abstracts |
| PROM or PREM in OSA-relevant outcome domain reported | Studies reporting on pediatric populations |

**Supplementary table S1: Inclusion and exclusion criteria**

1. **Risk of bias assessment**

| **First author** | **Year** | **Bias due to confounding** | **Bias due to selection of participants** | **Bias in classification of interventions** | **Bias due to deviations from intended interventions** | **Bias due to missing data** | **Bias in measurement of outcome** | **Bias in selection of reported results** | **Overall bias** |
| --- | --- | --- | --- | --- | --- | --- | --- | --- | --- |
| Baptista et al. | 2022 | ● moderate | ● moderate | ● low | ● low | ● serious | ● low | ● low | ● critical |
| Eastwood PR et al. | 2011 | ● low | ● moderate | ● low | ● low | ● serious | ● low | ● low | ● serious |
| Eastwood PR et al. | 2019 | ● low | ● serious | ● low | ● low | ● low | ● low | ● low | ● moderate |
| Heiser C et al. | 2017 | ● low | ● low | ● low | ● low | ● serious | ● low | ● low | ● moderate |
| Heiser C et al. | 2022 | ● low | ● low | ● low | ● low | ● low | ● low | ● low | ● low |
| Hinder D et al. | 2022 | ● low | ● low | ● low | ● low | ● serious | ● low | ● low | ● moderate |
| Hofauer B et al. | 2017 | ● low | ● low | ● low | ● low | ● serious | ● low | ● low | ● moderate |
| Hofauer B et al. | 2019 | ● low | ● low | ● low | ● low | ● moderate | ● low | ● low | ● low |
| Huntley C et al. | 2018 | ● low | ● low | ● low | ● low | ● moderate | ● low | ● low | ● low |
| Kent DT et al. | 2019 | ● low | ● low | ● low | ● low | ● low | ● low | ● low | ● low |
| Kent DT et al. | 2016 | ● low | ● serious | ● low | ● low | ● serious | ● low | ● low | ● serious |
| Kezirian EJ et al. | 2014 | ● low | ● low | ● low | ● low | ● serious | ● low | ● low | ● moderate |
| Kumar AT et al. | 2020 | ● low | ● low | ● low | ● low | ● moderate | ● low | ● low | ● low |
| Lee CH et al. | 2019 | ● low | ● low | ● low | ● low | ● serious | ● low | ● low | ● moderate |
| Mahmoud AF et al. | 2018 | ● low | ● low | ● low | ● low | ● serious | ● low | ● low | ● moderate |
| Mwenge GB et al. | 2013 | ● low | ● serious | ● low | ● low | ● low | ● low | ● low | ● moderate |
| Parikh V a et al. | 2018 | ● low | ● moderate | ● low | ● low | ● serious | ● low | ● low | ● serious |
| Pascoe M et al. | 2022 | ● low | ● low | ● low | ● low | ● serious | ● low | ● low | ● moderate |
| Patil R et al. | 2021 | ● low | ● low | ● low | ● low | ● serious | ● low | ● low | ● moderate |
| Patil R et al. | 2021 | ● low | ● low | ● low | ● low | ● serious | ● low | ● low | ● moderate |
| Pawlak D et al. | 2021 | ● low | ● low | ● low | ● low | ● serious | ● low | ● low | ● moderate |
| Philip P et al. | 2018 | ● low | ● serious | ● low | ● low | ● serious | ● low | ● low | ● critical |
| Sarber KM et al. | 2020 | ● low | ● low | ● low | ● low | ● serious | ● low | ● low | ● moderate |
| Sarber KM et al. | 2020 | ● low | ● moderate | ● low | ● low | ● serious | ● low | ● low | ● serious |
| Shah J et al. | 2018 | ● low | ● low | ● low | ● low | ● serious | ● low | ● low | ● moderate |
| Steffen A et al. | 2019 | ● low | ● low | ● low | ● low | ● serious | ● low | ● low | ● moderate |
| Steffen A et al. | 2020 | ● low | ● low | ● low | ● low | ● serious | ● low | ● low | ● moderate |
| Suurna M et al. | 2021 | ● low | ● low | ● low | ● low | ● serious | ● low | ● low | ● moderate |
| Van de Heyning PH et al. | 2012 | ● low | ● low | ● low | ● low | ● serious | ● low | ● low | ● moderate |
| Weeks B et al. | 2018 | ● low | ● moderate | ● low | ● low | ● serious | ● low | ● low | ● serious |
| Withrow K et al. | 2019 | ● low | ● low | ● low | ● low | ● low | ● low | ● low | ● low |
| Zhu Z et al. | 2018 | ● low | ● low | ● low | ● low | ● serious | ● low | ● low | ● moderate |

**Supplemenetary table S2: Risk of bias assessment of included observational and comparative studies using the ROBINS-I tool**

| **First author** | **Year** | **Bias from randomization process** | **Bias from deviations from intended interventions** | **Bias from missing outcome data** | **Bias from measurement of the outcome** | **Bias from selection of the reported results** | **Overall bias** |
| --- | --- | --- | --- | --- | --- | --- | --- |
| Heiser C et al. | 2021 | ● low | ● low | ● low | ● low | ● low | ● low |
| Woodson BT et al. | 2018 | ● low | ● low | ● low | ● low | ● low | ● low |

**Supplementary table S3: Risk of bias assessment of included randomized controlled studies using the Risk-of-bias 2 tool**

1. **Publication bias assessment**

**Supplementary figure S1: Funnel plot before trim and fill adjustment**

**Supplementary figure S2: Funnel plot after trim and fill adjustment**
